# Using socially distanced and online simulation training to improve the confidence of junior doctors in psychiatry

**DOI:** 10.1192/bjb.2022.18

**Published:** 2023-08

**Authors:** Thomas Hewson, Hayley Foster, Ruth Sanderson

**Affiliations:** 1 Pennine Care NHS Foundation Trust, UK; 2 University of Manchester, UK

**Keywords:** Education and training, simulation, junior doctor, medical education, psychiatry

## Abstract

**Aims and method:**

The authors designed and delivered simulation training to improve the confidence and competence of junior doctors beginning work in psychiatry. Junior doctors completed various simulated psychiatry scenarios while receiving personalised feedback and teaching from their peers in online or socially distanced settings. Learners rated their confidence in psychiatry skills pre- and post-session, and Wilcoxon signed-rank tests were conducted to detect statistically significant differences. Qualitative feedback was analysed thematically.

**Results:**

Twenty-one junior doctors attended the training. There were statistically significant (*P* < 0.05) improvements in trainee confidence across all psychiatry skills tested. The most enjoyable aspects of the session included its ‘interactivity’, relevance to clinical practice, and ‘realistic’ and ‘interesting’ simulated scenarios.

**Clinical implications:**

Near-peer simulation teaching, delivered both in person and online, is effective at improving junior doctors’ confidence in psychiatry. Delivering this training during placement induction could help to ensure adequate preparation of, and support for, new doctors.

## Introduction

Psychiatry is a challenging and rewarding specialty that provides doctors with transferable skills for many careers. In 2014, Health Education England (HEE) recommended that at least 22.5% of Foundation Year 1 (FY1) doctors and 22.5% of Foundation Year 2 (FY2) doctors complete 4-month rotations in psychiatry.^1^ General practitioner (GP) trainees also regularly complete clinical posts in the specialty, with mental health being a key component of the Royal College of General Practitioners curriculum.^2^

It is important both for trainee well-being and patient safety that all doctors working in psychiatry feel adequately prepared and supported. Ensuring positive and supportive experiences of postgraduate psychiatry are also essential to recruitment and retention. For example, work experience is a major determinant of career choice, and most decisions to pursue psychiatry occur following graduation.^3–6^ In particular, requirements to practice independently during on-call shifts and access senior supervision remotely out of hours means that junior doctors must feel confident managing acute psychiatry presentations and emergencies; this contrasts to medical and surgical specialties, where senior support for foundation and core trainee doctors is typically readily available on-site.^7^

Prior research has demonstrated that many junior doctors perceive themselves to lack knowledge, skills and confidence for assessing mental health patients.^8,9^ Holt et al found that 63% of first-year core psychiatry trainees were ‘neutral’ or ‘disagreed’ that they felt prepared for on-call shifts in the specialty.^9^ This may be partly because of the wide variation in coverage of psychiatry at UK medical schools and/or a lack of recent exposure to the specialty.^10^ For foundation trainees, the transition from student to doctor can also present additional challenges and represent a time of stress and low confidence, emphasising their need for support, training and effective placement induction.^11,12^

### Simulation training

Simulation training is rooted in experiential learning theory, encouraging people to learn through reflection on their performance in different clinical contexts.^13^ This teaching method can provide a safe environment for learners to recognise their weaknesses and learn from mistakes, without the risk of harming or distressing patients.^14^ Simulation training has proven effectiveness for developing communication skills among psychiatry trainees and preparing doctors for challenging on-call responsibilities.^7,15–17^ A recent meta-analysis of simulation training in psychiatry found significant improvements in the knowledge, skills, attitudes and behaviours of medical learners, as well as benefits for patients.^17^

### Aims and objectives

The authors aimed to design and deliver a simulation-based training session for foundation doctors, GP trainees and core psychiatry trainees rotating into psychiatry placements. As a result of the COVID-19 pandemic and resulting requirements to provide socially distanced and/or online teaching, the authors explored whether simulation training was feasible and effective when delivered online and when using peers as both facilitators and role-play actors.

## Method

### Design of the training session

Core and higher psychiatry trainees, led and supported by a consultant psychiatrist and the postgraduate tutor (person responsible for the organisation and delivery of postgraduate psychiatry education in the local hospital), planned a simulation-based training session for junior doctors rotating into psychiatry. This group of stakeholders discussed frequently encountered and/or high-pressure situations faced during on-call shifts in the specialty, and the skills required for confidently and safely managing these. Several simulated scenarios were subsequently created to teach and assess these skills and performance in these situations. Content validity was assured through pilot testing of the scenarios with two core psychiatry trainees and one GP trainee working the local psychiatry on-call rota.

Five simulated scenarios were created covering the following skills: identification, assessment and management of alcohol withdrawal; psychiatric history-taking; clinical risk assessment (including risk of suicide); understanding of Section 136 of the Mental Health Act 1983 and assessment of a patient detained under this Section; use and implementation of Section 5(2) of the Mental Health Act 1983; management of physical health problems in comorbid elderly patients with cognitive impairment; and identification, assessment and management of side-effects of antipsychotic medications. Management of acute agitation and violence was also highlighted as an important skill in psychiatry; however, this topic was taught didactically because of the difficulties associated with role-playing aggression.

**Fig. 1:**
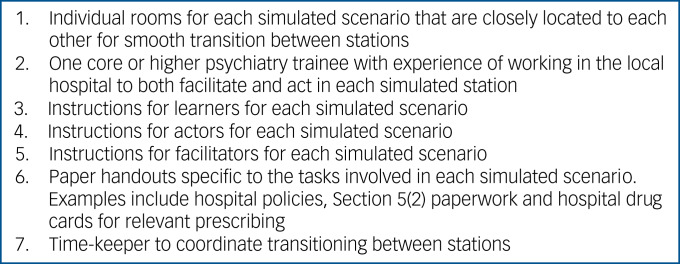
List of resources required for face-to-face on-call simulation training sessions.

Several resources were required for the training, all of which were readily available (Fig. 1). Five rooms in close proximity to each other were used for conducting individual simulated scenarios. Room sizes were large enough to enable social distancing between session facilitators and learners, with just one facilitator and learner in each room. Session facilitators were core or higher psychiatry trainees with experience working out-of-hours in the local hospital, thus encouraging near-peer learning and support, as well as promoting discussion about local on-call procedures. The role of the session facilitators included providing feedback to learners and teaching specific to individual scenarios, as well as showing them how to navigate relevant clinical guidelines and policies. Because of social distancing precautions and the need to limit the number of people in each room, the core or higher psychiatry trainee facilitating each scenario also acted as the simulated patient. Paper-based activities and handouts were provided for each station, which included relevant hospital policies, Section 5(2) paperwork and drug charts for prescribing tasks.

### Delivery of the training session

The simulation training was delivered to two groups of foundation doctors (involving both FY1 and FY2 doctors) and one mixed group of GP trainees and core psychiatry trainees beginning work at a local psychiatry service. Both sessions for foundation doctors occurred face to face, following social distancing precautions, whereas the session for GP and psychiatry trainees was held online because of the evolving COVID-19 restrictions at the time. The training occurred within the third week of placement for the first group of foundation trainees, because of busy induction schedules; however, based on initial feedback, the session was subsequently moved and delivered during the first week of induction for other trainees.

Learners were briefed and given instructions on how the workshop would be conducted before rotating around all five simulated scenarios. Pendleton's rules were followed to provide individualised feedback to learners at the end of each scenario. Applying these rules, facilitators initially clarified relevant theory and then encouraged trainees to identify their individual strengths, before discussing their own observations of what the trainee did well. Learners were then prompted to identify individual areas of development and how they could have improved their performance in managing each simulation, with the facilitator subsequently providing their own perspectives. Finally, facilitators and trainees jointly discussed action plans to meet any identified learning needs and to further improve relevant skills.^18^ To reduce learner anxiety and create a relaxed learning atmosphere, no specific marking or scoring occurred, and all feedback was delivered verbally and informally alongside teaching. Group debriefing occurred once all trainees had completed all scenarios. Common strengths and areas for development were reviewed across the cohort, and further teaching was provided on relevant content, including answering group questions.

In total, the session lasted for approximately 2 hours. Pre-briefing occurred for 15 min, each individual scenario lasted for 15 min (including 5–7 min of personalised feedback and teaching) and debriefing lasted for 30 min. These timings were selected to achieve a balance between the depth and breadth of clinical simulations covered, while being mindful of the time constraints of the wider induction programme.

The online version of the workshop was delivered remotely, using Zoom for macOS, version 5.4.9 (59931.0110; Zoom Video Communications Inc., San Jose, CA, USA (https://zoom.us/download). The simulated scenarios were kept the same, but paper-based tasks such as prescribing and completing Section 5(2) paperwork were not physically completed by the trainees. Instead, all training materials were displayed electronically, using the ‘share screen’ function, and trainees verbalised how they would complete the written tasks before model answers were shared. Learners and session facilitators/actors kept their cameras switched on during the simulated scenarios to increase the fidelity of the simulation and teach nonverbal communication. All trainees worked in pairs to complete the online simulated scenarios instead of individually; this decision was made to encourage greater interaction between trainees in the online setting and because of a larger group size.

### Data collection

Feedback was collected by questionnaires that were completed by learners immediately before and after the training (see Supplementary Appendices 1 and 2, available at https://doi.org/10.1192/bjb.2022.18). Participation was voluntary and all participants gave informed consent to provide evaluation data. Ethical approval was not necessary since this was a training evaluation, which was confirmed by the local research and development department.

The questionnaires contained a combination of Likert scales and free-text responses. Likert scales were used to numerically rate learners’ confidence in skills relevant to psychiatry, ranging from 1 (not at all confident) to 5 (very confident). Free-text responses permitted the collection of qualitative data regarding learners’ experiences of the workshop and suggestions for improvement.

Quantitative data was exported into SPSS for macOS version 25.^19^ Median confidence ratings were calculated and compared before and after the training. Since the data was nonparametric and included paired observations, pre- and post-workshop Likert scale ratings of learner confidence were statistically analysed to detect significant differences by using Wilcoxon signed-rank tests. Free-text qualitative data were exported into Microsoft Excel for macOS version 16.45 and emergent themes were agreed and summarised from the data by the authorship team. Subgroup analyses were conducted comparing foundation doctors that received the training in person to GP trainees and core psychiatry trainees who engaged in the session remotely.

## Results

### Participants

Twenty-one learners participated in the workshop and provided feedback, giving a 100% response rate. The sample comprised 11 foundation doctors, seven GP trainees and three core psychiatry trainees.

### Quantitative data

Pre-workshop median confidence ratings for the various psychiatry skills and on-call scenarios varied from 1 (not at all confident) to 3 (neutral) (Table 1). Trainees reported the most confidence in managing physical health problems in psychiatry (median 3), and the least confidence in assessing patients detained under Section 136 of the Mental Health Act 1983 (median 1).

**Table 1:** Pre- and post-workshop confidence ratings by junior doctors

Skill	Pre-workshop median confidence rating (1–5)	Post-workshop median confidence rating (1–5)	% Difference in median confidence rating	Wilcoxon signed-rank test statistic (*Z*)	*P*-value
History-taking	3	4	+33.33%	−3.531	*P* < 0.001**
Risk assessment	3	4	+33.33%	−3.066	*P* = 0.002**
Section 136 assessment	1	3	+200%	−3.552	*P* < 0.001**
Use of Section 5(2)	2	4	+100%	−3.647	*P* < 0.001**
Physical health review	3	4	+33.33%	−3.274	*P* = 0.001**
Managing side-effects of antipsychotics	2	3	+50%	−3.611	*P* < 0.001**
Assessment and management of alcohol withdrawal	3	4	+33.33%	−3.656	*P* < 0.001**
Overall confidence working in psychiatry	2	3	+50%	−3.145	*P* = 0.002**

** *P* < 0.01.

After delivery of the workshop, there was a statistically significant improvement in the confidence of trainees across all psychiatry skills tested (Table 1). The percentage improvement in median confidence ratings ranged from 33.33 to 200%. The largest improvements in median confidence ratings were observed for trainees’ confidence in understanding and implementing Section 5(2) of the Mental Health Act 1983, and assessing patients detained under Section 136 of the Mental Health Act 1983. Post workshop median confidence ratings ranged from 3 (neutral) to 4 (very confident).

### Qualitative data

Nineteen respondents listed their favourite aspects of the workshop, and two respondents did not answer this question. Trainees most enjoyed the ‘interactive’ nature of the training (*n* = 5, 26.3%) and its helpfulness for aiding clinical practice (*n* = 5, 26.3%). Trainees frequently commented that the simulated scenarios were ‘realistic’ and involved ‘good’ or ‘interesting’ cases (*n* = 4, 21.1%). Session facilitators received praise for their enthusiasm and teaching ability (*n* = 4, 15.8%). Two trainees reported valuing individual feedback to help understand their strengths and areas for development before starting psychiatry placements. One trainee enjoyed the ‘opportunity to ask questions in a friendly setting’, and another thought that it was useful to hear the approaches and questions of their peers.

Only two respondents listed their least favourite aspects of the workshop. One trainee commented that they least enjoyed the ‘surprise OSCEs’. One trainee also commented that relevant clinical information could have been displayed on computer screens throughout each virtual simulated scenario.

Four trainees expressed a desire for more or similar sessions in the future, especially during placement induction. One trainee commented that the workshop was ‘a really good idea’, and another suggested including a simulated scenario addressing ‘rapid tranquilisation’ policies.

### Subgroup analyses

A statistically significant (*P* < 0.05) improvement was demonstrated in the confidence of foundation doctors across all psychiatry skills tested after the workshop (Table 2). Except for psychiatric risk assessment and assessment of physical health problems in elderly patients with cognitive impairment, a statistically significant (*P* < 0.05) improvement was also demonstrated in the confidence of GP trainees and core psychiatry trainees across all psychiatry skills tested after the workshop (Table 3).

**Table 2:** Subgroup analysis of pre- and post-workshop confidence ratings by foundation doctors

Skill	Pre-workshop median confidence rating (1–5)	Post-workshop median confidence rating (1–5)	% Difference in median confidence rating	Wilcoxon signed-rank test statistic (*Z*)	*P*-value
History-taking	3	4	+33.33%	−2.873	*P* = 0.004**
Risk assessment	3	4	+33.33%	−2.754	*P* = 0.006**
Section 136 assessment	1	4	+300%	−2.971	*P* = 0.003**
Use of Section 5(2)	1	4	+300%	−2.994	*P* = 0.003**
Physical health review	3	4	+33.33%	−2.807	*P* = 0.005**
Managing side-effects of antipsychotics	2	4	+100%	−2.859	*P* = 0.004**
Assessment and management of alcohol withdrawal	3	4	+33.33%	−2.992	*P* = 0.003**
Overall confidence working in psychiatry	2	3	+50%	−2.428	*P* = 0.015*

* *P* < 0.05, **P < 0.01.

**Table 3:** Subgroup analysis of pre- and post-workshop confidence ratings by general practitioner specialist trainees and core psychiatry trainees

Skill	Pre-workshop median confidence rating (1–5)	Post-workshop median confidence rating (1–5)	% Difference in median confidence rating	Wilcoxon signed-rank test statistic (*Z*)	*P*-value
History-taking	3	3.50	+16.67%	−2.121	*P* = 0.034*
Risk assessment	3	3.50	+16.67%	−1.414	*P* = 0.157
Section 136 assessment	1.5	3	+100%	−2.070	*P* = 0.038*
Use of Section 5(2)	2.5	3.50	+40%	−1.994	*P* = 0.046*
Physical health review	3	3.50	+16.67%	−1.732	*P* = 0.083
Managing side-effects of antipsychotics	3	3	0	−2.333	*P* = 0.020*
Assessment and management of alcohol withdrawal	3	3	0	−2.236	*P* = 0.025*
Overall confidence working in psychiatry	2.50	3.50	+40%	−2.070	*P* = 0.038*

* *P* < 0.05.

GP trainees and core psychiatry trainees generally reported greater confidence in skills in psychiatry compared with foundation doctors before the workshop; however, this trend reversed, and greater confidence ratings were generally reported by foundation doctors following the training. The percentage difference in median confidence ratings before and after the workshop ranged from 33.3 to 300% for foundation doctors, and 0 to 100% for GP and core psychiatry trainees.

## Discussion

Our results indicate that near-peer simulation training is an effective and valuable method for improving the confidence and skills of junior doctors beginning work in psychiatry. Furthermore, simulation training can effectively be delivered and positively received in online settings, and when applying social distancing precautions with a sole person behaving as both a simulated patient and scenario facilitator.

Besides from the positive evaluation data, there were various benefits to the training. The workshop was low-cost, required minimal resources and training materials were easily replicated between sessions. The simulation workshop was also easily delivered online with no technical difficulties, allowing medical education and peer learning to continue during the COVID-19 pandemic. Utilising core and higher psychiatry trainees as session facilitators provided the advantage of site-specific advice being delivered during the training, because of their current or prior experiencing of working on-call in the local hospital; this advice included how and where to access local hospital policies, how to elicit senior support and follow local reporting procedures, and how to address clinical demands typically encountered in the local context.

Peer-led teaching is associated with improved retention and application of knowledge, learner motivation and academic performance, compared with lecture-style formats.^20–22^ This was echoed in comments by some trainees that the workshop was preferable to didactic induction presentations. Peer teaching can also create a relaxed and friendly learning atmosphere, encouraging discussion of sensitive topics, networking and socialisation.^20,23^ These benefits of peer teaching extend to trainees delivering training, providing them with opportunities for leadership and communication skills development.^20,23^ Acting as simulated patients may help trainees to improve their own clinical skills, with clinical role-play promoting reflection and insight, and increasing a person's empathetic abilities.^24^ To maximise the benefits of junior doctors acting as teaching facilitators, Till et al suggested that workplace-based assessments can be completed by seniors observing their simulation teaching;^7^ this was not possible in the current project because of room sizes and social distancing precautions, in the context of COVID-19, but represents a potential area for future development.

Interestingly, less significant improvements in confidence were seen for GP and core psychiatry trainees accessing the training online compared with foundation doctors receiving face-to-face education. This may be because GP and core psychiatry trainees generally entered the training with higher reported self-confidence, therefore having less room for improvement. Alternatively, the online format may have been less effective than face-to-face education, although only one trainee highlighted difficulties with virtual training. McCutcheon et al reviewed the effect of online and face-to-face learning of clinical skills in nursing education and concluded similar effectiveness.^25^ Furthermore, online videos of simulated patient cases have previously been found to provide learner satisfaction and improved confidence in psychiatry;^26^ however, there is a lack of research directly comparing online and face-to-face simulation teaching in the specialty.

Since the establishment of the training session, it has become a regular part of the junior doctor's induction at the local psychiatry hospital. In the future, the training will be extended to include additional simulated scenarios. For example, prior literature has demonstrated the effective use of simulation to teach other emergency psychiatry topics, including assessing suicide risk in accident and emergency departments, negotiating community treatment plans, psychotropic prescribing, seclusion reviews, managing delirium, managing oversedation, and identifying and managing lithium toxicity.^7,15,27^ There may additionally be scope to deliver the workshop to a wider audience, including foundation doctors not currently working in psychiatry. This could be useful for several reasons. First, foundation doctors must demonstrate evidence of a broad range of competencies across several specialties, including mental health for revalidation.^28^ The importance of mental health training and skills development for all foundation doctors is also emphasised in the recent HEE Foundation Programme Review.^29^ Furthermore, because of the high prevalence of psychiatric comorbidities in medical, surgical and primary care settings,^30–33^ many of the simulated scenarios will be encountered by doctors practicing outside of psychiatry.

There are several limitations to this training evaluation. First, the small sample size of 21 doctors limits the generalisability of our results to the wider junior doctor population. However, the small sample size did not prevent the demonstration of several statistically significant outcomes, indicating sufficient statistical power of the study. Second, the participant subgroups of foundation doctors, GP trainees and core psychiatry trainees were unequally split, and only three core psychiatry trainees took part in the evaluation; this makes it difficult to draw definitive conclusions about differential effects of the training on different junior doctor grades and specialties. Third, the prior psychiatry experience of doctors partaking in the training was not recorded, and the accuracy of trainee's self-reported confidence ratings pre- and post- session are uncertain. For example, prior research has demonstrated poor accuracy of doctors’ self-assessment compared with externally observed measures of performance.^34^

Future research should evaluate the experiences of trainees acting as simulation facilitators, and the longer-term effects of simulation training in psychiatry. Applying Kirkpatrick's four-stage evaluation model, future researchers should explore the effects of face-to-face and online psychiatry simulation training on junior doctor's behaviour in clinical settings and patient outcomes.^35,36^ Evaluating the benefits and challenges of simulation training across larger samples of junior doctors, including doctors from different hospital trusts and of different training grades, would improve the generalisability of future studies. The effects of longer and shorter clinical simulations, as well as involving professional actors and expert patients in the delivery of simulation training during placement inductions, additionally warrants future consideration.

In conclusion, our project has demonstrated several benefits of providing near-peer simulation training, both in person and online, for junior doctors in psychiatry. Mental health trusts should consider including such training methods in junior doctor's inductions to ensure adequate preparation, confidence and support for working in the specialty. Further research is needed to explore the longer-term effects and relative effectiveness of different simulation training methods.

## Data Availability

The data that support the findings of this study are available from the corresponding author, [TH], upon reasonable request.

## About the authors

**Thomas Hewson** (BMBS, BMedSci (Hons)) is a Core Trainee in Psychiatry with Pennine Care NHS Foundation Trust, UK; and an Academic Clinical Fellow in Psychiatry at the, University of Manchester, UK. **Hayley Foster** (MBChB) is a Core Trainee (CT3) in Psychiatry with Pennine Care NHS Foundation Trust, UK. **Ruth Sanderson** (MBChB, BSc) is a Consultant Psychiatrist in Old Age Psychiatry and Postgraduate Tutor in Psychiatry with Pennine Care NHS Foundation Trust, UK.

## Supplementary material

For supplementary material accompanying this paper visit https://doi.org/10.1192/bjb.2022.18. click here to view supplementary material
