# Supplementary material for: Using socially distanced and online simulation training to improve the confidence of junior doctors in psychiatry
Source: BJPsych Bull. 2023 Aug;47(4):235–41. doi: 10.1192/bjb.2022.18 (PMC10387411; doi:10.1192/bjb.2022.18)
Supplement: Supplementary file 1 [file S2056469422000183sup001.docx]

**“Using Socially Distanced and Online Simulation Training to Improve the Confidence of Junior Doctors in Psychiatry” - Supplementary Files**

**Appendix 1: Pre-workshop training evaluation survey**

We are collecting feedback regarding the workshop to evaluate this training session and improve the teaching for future cohorts. Anonymised feedback data may be presented at relevant medical education conferences and/or published in a peer-reviewed journal.

Please indicate below whether you consent to the completion of this survey and the above uses of your anonymised data:

Yes No

Please indicate your level of training:

For the remaining questions, please rate your confidence in completing the following tasks and skills on a scale of 1 (not at all confident) to 5 (very confident). Please circle your selected ratings.

**Psychiatry history taking**

1 2 3 4 5

Not at all confident Somewhat confident Very confident

**Psychiatry risk assessment**

1 2 3 4 5

Not at all confident Somewhat confident Very confident

**Assessment and management of a patient in the Section 136 suite**

1 2 3 4 5

Not at all confident Somewhat confident Very confident

**The use and implementation of Section 5(2) of the MHA**

1 2 3 4 5

Not at all confident Somewhat confident Very confident

**Assessment and management of physical health problems in psychiatry**

1 2 3 4 5

Not at all confident Somewhat confident Very confident

**Management of side effects of psychiatric medications**

1 2 3 4 5

Not at all confident Somewhat confident Very confident

**Assessment and management of substance misuse and withdrawal**

1 2 3 4 5

Not at all confident Somewhat confident Very confident

**Overall confidence in completing common and emergency tasks in psychiatry:**

1 2 3 4 5

Not at all confident Somewhat confident Very confident

**Appendix 2: Post-workshop training evaluation survey**

For the below questions, please rate your confidence in completing the following tasks and skills on a scale of 1 (not at all confident) to 5 (very confident). Please circle your selected ratings.

**Psychiatry history taking**

1 2 3 4 5

Not at all confident Somewhat confident Very confident

**Psychiatry risk assessment**

1 2 3 4 5

Not at all confident Somewhat confident Very confident

**Assessment and management of a patient in the Section 136 suite**

1 2 3 4 5

Not at all confident Somewhat confident Very confident

**The use and implementation of Section 5(2) of the MHA**

1 2 3 4 5

Not at all confident Somewhat confident Very confident

**Assessment and management of physical health problems in psychiatry**

1 2 3 4 5

Not at all confident Somewhat confident Very confident

**Management of side effects of psychiatric medications**

1 2 3 4 5

Not at all confident Somewhat confident Very confident

**Assessment and management of substance misuse and withdrawal**

1 2 3 4 5

Not at all confident Somewhat confident Very confident

**Overall confidence in completing common and emergency tasks in psychiatry:**

1 2 3 4 5

Not at all confident Somewhat confident Very confident

Please comment on your favourite aspects of the on-call psychiatry workshop:

______________________________________________________________________________________________________________________________________________________

___________________________________________________________________________

Please comment on your least favourite aspects of the on-call psychiatry workshop:

______________________________________________________________________________________________________________________________________________________

___________________________________________________________________________

Please use this space for any further comments or suggestions about the training:

______________________________________________________________________________________________________________________________________________________

___________________________________________________________________________
